# Approximate measurement invariance in cross-classified rater-mediated assessments

**DOI:** 10.3389/fpsyg.2014.01469

**Published:** 2014-12-23

**Authors:** Ben Kelcey, Dan McGinn, Heather Hill

**Affiliations:** ^1^College of Education, Criminal Justice and Human Services, University of CincinnatiCincinnati, OH, USA; ^2^Graduate School of Education, Harvard UniversityCambridge, MA, USA

**Keywords:** measurement invariance, random item effects, multilevel item response models, teaching, measurement equivalence

## Abstract

An important assumption underlying meaningful comparisons of scores in rater-mediated assessments is that measurement is commensurate across raters. When raters differentially apply the standards established by an instrument, scores from different raters are on fundamentally different scales and no longer preserve a common meaning and basis for comparison. In this study, we developed a method to accommodate measurement noninvariance across raters when measurements are cross-classified within two distinct hierarchical units. We conceptualized random item effects cross-classified graded response models and used random discrimination and threshold effects to test, calibrate, and account for measurement noninvariance among raters. By leveraging empirical estimates of rater-specific deviations in the discrimination and threshold parameters, the proposed method allows us to identify noninvariant items and empirically estimate and directly adjust for this noninvariance within a cross-classified framework. Within the context of teaching evaluations, the results of a case study suggested substantial noninvariance across raters and that establishing an approximately invariant scale through random item effects improves model fit and predictive validity.

The use of rater inferential judgment is a common and persistent feature of assessments designed to measure latent constructs across many different fields of research (e.g., Engelhard, 2002). In these types of assessments, raters typically conduct evaluations by interpreting evidence (e.g., responses, behaviors) using their trained, but subjective, judgments. For this reason, the use of raters to assign scores has been described as an indirect or rater-mediated process because measurements are not directly observed but rather inferred through raters' judgments (Bejar et al., 2006).

An important assumption underlying meaningful comparisons in rater-mediated assessments is that measurement is invariant across raters. Measurement invariance across raters suggests that raters use items similarly so that the relationships between a latent trait and the manifest items with which it is measured do not depend upon which rater conducted an evaluation^1^. When items function differently across raters, ratings no longer preserve a common meaning and basis for comparison across raters because scales are rater-specific. In this way, the extent to which a common scale can be formed across raters depends largely on the extent to which raters share a common basis for assigning scores.

Research has shown that a significant source of construct-irrelevant variation in many rater-mediated assessments arises from differences among raters in how they apply the standards established by an instrument (e.g., Hill et al., 2012). Although findings of rater differences are not surprising, the magnitude and item-specific nature of these differences found by recent reports have demonstrated just how critical of an issue rater variability can be and raises questions about the degree to which scores from different raters are on commensurate scales (Kane and Staiger, 2012). Despite extensive and consistent evidence of rater differences across a broad array of assessments, scores from different raters are routinely treated as if they were exchangeable across raters and are often used to make high-stakes comparative decisions (e.g., Baumgartner and Steenkamp, 2001; Engelhard, 2002; Linacre and Wright, 2002; Eckes, 2009a,b; Schochet and Chiang, 2010; Kane and Staiger, 2012).

In this study, we developed a method to accommodate measurement noninvariance across raters when measurements are nested within raters and (optionally) cross-classified among other distinct hierarchical units (e.g., countries). To do so, we extend cross-classified (multilevel) graded response models to incorporate random item (discrimination and threshold) effects to test, calibrate, and account for measurement noninvariance among raters. By leveraging empirical estimates of rater-specific deviations in the item parameters, the proposed method affords identification of noninvariant items and empirical estimation and direct adjustment for noninvariance within a multilevel or cross-classified framework.

To explore the value of the approach, we applied the proposed method to a case study of repeated classroom measures of teaching quality using three primary questions. First, we investigated the extent to which there was evidence of measurement noninvariance among raters in cross-classified rater-mediated assessments of teaching. Second, we examined the extent to which allowing item parameters to vary across raters improved the relative and absolute fit of the measurement model as compared to models that assume invariant item parameters. Finally, because a primary criterion for the validity of classroom observations is their efficacy in predicting student achievement gains, we assessed the extent to which allowing item parameters to vary across raters improved the predictive efficacy of observation scores as compared to more conventional approaches.

## Background

### Rater-mediated assessments

Raters have played a critical role in evaluating a wide range of psychological, cognitive, and physical traits. For example, teachers have been used as raters to assess students' medication use and deviant behavior (Conners, 1969; Werry et al., 1975); teachers have been used to rate children's levels of hyperactivity (Gordon, 1979); college instructors have been used to rate students' writing quality (Sudweeks et al., 2004); school principals or trained raters have been used to describe and evaluate teaching through portfolios, instructional diaries, and classroom observations (Brophy, 1986; Kane and Staiger, 2012).

The impetus for the use of rater-mediated assessments stems largely from the position that they often allow for more authentic and relevant assessments, thereby improving support for the validity of an assessment. Despite the flexibility and authenticity offered by rater-mediated assessments, they are often paired with features that, without proper treatment, can undermine their validity and reliability. In particular, a key threat to their validity is the construct-irrelevant variance introduced by the differences among raters in how they award scores (Messick, 1989).

Research across multiple disciplines has demonstrated that such differences manifest in a number of common ways. Perhaps the most commonly cited rater effect is the differences among raters in terms of the severity with which they apply their evaluations. Differences in severity occur when some raters provide ratings that are consistently more severe relative to other raters (Linacre and Wright, 2002). More complex differences of this type can also take root when, for example, rater severity varies across items and/or categories within items. For instance, for a given item some raters may perceive the implied proficiency levels of two adjacent ratings to be further apart than other raters do (Eckes, 2009b). Other common rater effects include a halo effect and a central/extreme tendency effect. Rater halo effects can occur when raters place undue emphasis on a specific competency (Engelhard, 2002). Central/extreme tendencies manifest when raters avoid or use only the extreme categories of a scale (Baumgartner and Steenkamp, 2001).

Together these and other inconsistencies across raters potentially introduce measurement noninvariance because the categories of a scale may no longer have a consistent meaning across raters. Left untreated, rater noninvariance has the potential to unfairly affect outcomes and undermine the reliability and validity of rater-mediated assessments (Messick, 1989).

### Analysis of rater-mediated assessments

There are a wide variety of approaches to analyzing rater-mediated assessments (e.g., Baumgartner and Steenkamp, 2001; Engelhard, 2002; Patz et al., 2002; Wolfe, 2004; Bejar et al., 2006; De Jong et al., 2007; Lahuis and Avis, 2007; Hill et al., 2012; Carlisle et al., 2013). We focus our discussion on one common treatment of rater-mediated assessments that draws on multilevel measurement models to track rater differences through random effects (e.g., Lahuis and Avis, 2007). We consider two general data structures that are relevant to the proposed model and conceptually outline the application of multilevel measurement models to these data structures.

#### Hierarchically nested assessments

In studies focused on the comparative evaluation of individuals (e.g., examinees, respondents), assessments are often obtained through the judgmental scoring of participants on targeted indicators (e.g., behaviors, responses) by individual judges. The structure of this design is often considered to have a multilevel organization because participants are hierarchically nested within raters. As previously noted, an important implication of this design is that, to the extent that raters vary in their application of the instrument standards, participants judged by the same rater share construct-irrelevant variation owing to differences among raters. As a result, the nested structure of this design potentially confounds variation in the underlying construct with differences among raters because variation in awarded scores incorporates variation owing to both of these components.

Because the goal of rater-mediated assessments is to assess participants free of rater influence, research has accounted for rater differences by introducing rater effects through, for example, a multilevel item response theory framework (e.g., Lahuis and Avis, 2007). For instance, using an item response model (IRM) where items are incorporated as fixed effects, associations among items are decomposed into a component due to the targeted latent trait and a component designed to capture persistent differences among raters in terms of their relative severity across all items. Given dichotomous items, we might express the probability of receiving a rating of one on item *i* in for participant *t* rated by rater *r* as following a multilevel IRM (where Φ is the normal cumulative distribution function).

(1) $$P(Y_{itr}=1)=\Phi (a_{i}\theta_{t}+a_{i}\gamma_{r}-d_{i})$$

Here, the probability of obtaining a one on an item is specified as a function of the level of the targeted construct for participant *t*, θ_*t*_, and the severity of the assigned rater, γ_*r*_, with associated item parameters, *a*_*i*_ as the discrimination parameter and *d*_*i*_ as the threshold parameter. Both latent variables are generally assumed to have a normal distribution and the scale can be set by fixing the distribution of θ_*t*_ ~ *N*(0, 1).

#### Cross-classified assessments

Separate from the nesting of participants in raters, rater-mediated assessments frequently introduce, or sustain other design features that further contribute to construct-irrelevant variance. For instance, repeated measures designs are often purposefully employed in conditions where measurement is known to be unreliable or sensitive to context (Hill et al., 2012). Similarly, many measurement designs operate within larger multilevel structures. For example, participants may be nested within schools or nested within countries (Steenkamp and Baumgartner, 1998; Fox, 2010).

A common result of these design features is that they introduce a cross-classified dependence structure in the data because each participant or observation is simultaneously nested within a rater and a second distinct non-hierarchical unit (Baayen et al., 2008). For example, under a repeated measures design, each participant is observed across multiple observations and each observation is rated by a different rater. Observations are thus nested within or cross-classified among participants and raters.

Under the repeated measures design, research has found evidence that scores among items within the same observation are likely to display excess variance arising from rater differences and idiosyncratic features of an observation (e.g., participant had a bad day). Because such excess variance is specific to an observation and rater and does not generalize beyond a sampled observation and rater, research has accounted for these effects by introducing observation- and rater-specific random effects (e.g., Carlisle et al., 2013). The introduction of random effects for each mode of the distinct hierarchies gives rise to a cross-classified (multilevel) IRM. Variation in the targeted latent trait is now decomposed into three components: a targeted participant component which persists across observations, an observation-specific component, and a rater component. Extending the multilevel IRM in Equation (1), we can now express the probability of obtaining a particular rating as

(2) $$P(Y_{iotr}=1)=\Phi (a_{i}\theta_{t}+a_{i}\alpha_{ot}+a_{i}\gamma_{r}-d_{i})$$

Equation (2) follows the aforementioned notation but now expands to accommodate (a) repeated measurements such that *Y*_*iotr*_ is the score on item *i* in observation *o* for participant *t* rated by rater *r* and (b) observation-specific deviations for observation *o* in participant *t* (α_*ot*_).

### Approaches to measurement invariance

To assess and substantiate invariance in these applications or correct for noninvariance, there have been three typical approaches: full, partial, and approximate invariance. Below we briefly outline their structure, application, and limitations as they may apply to rater-mediated assessments.

#### Full invariance

The conventional approach to assessing/establishing invariance across subgroups is through multiple group analyses. For instance, continuing with the aforementioned notation from the repeated measures cross-classified model (2), full invariance across raters supports

(3) $$P(Y_{iotr}=1|R,\;\theta )=P(Y_{iotr}=1|\theta )$$

(Mellenbergh, 1989). Put differently, for participants with the same level of the latent trait, the probabilities of a particular score on an item should not depend on which rater rated an observation (Millsap and Everson, 1993).

#### Partial invariance

Measurement becomes noninvariant when the relationships between a latent trait and items depend on which group an observation belongs to [e.g., the equality in Equation (3) no longer holds]. When there is evidence of measurement noninvariance, a common alternative approach is to adjust for noninvariant items using a partial measurement invariance approach (Steenkamp and Baumgartner, 1998). With partial measurement invariance, multiple group (e.g., rater-specific) measurement models are estimated and linked to form a common scale (across groups) by capitalizing on items that are invariant across all groups (i.e., anchor items). Despite the potential of the partial measurement invariance approach, literature has highlighted several important limitations (e.g., Holland and Wainer, 1993; Vandenberg, 2002; Steinmetz, 2013). Perhaps most germane to multilevel and cross-classified rater-mediated assessments is that empirical application of a partial invariance approach requires invariant items across all groups in order to bridge groups-specific scales. Lacking invariant items to anchor the scale across raters, multigroup partial invariance approaches are poorly suited to establish a common scale across groups (e.g., Holland and Wainer, 1993). Furthermore, even if two invariant items existed, estimating and testing for such invariance with a multigroup model would conceptually require estimating a separate measurement model for each rater. Given a large number of raters, stable estimation of item parameters would likely require large sample sizes and be computationally demanding because of the number of estimated parameters.

#### Approximate measurement invariance

When full or partial measurement invariance is intractable, a more flexible approach recently developed is to accommodate measurement noninvariance through hierarchically defined random item effects (Fox, 2010; Rijmen and Jeon, 2013). The prototypical application involves cross-national comparisons of latent traits with respondents nested within countries (Fox, 2010). To facilitate cross-national comparisons, measurement invariance requires items to function similarly in each country. When items are not invariant across countries, the approximate measurement invariance approach uses random item effects to model the extent to which item parameters vary across countries. This approach establishes an international measurement scale across countries using the mean of item parameters across all countries. Country-specific noninvariance in item parameters is then conceptualized as deviations from the international item parameters and captured through country-specific random item effects.

There are two primary practical advantages to this framework. First, in theory, a common scale can be established and cross-group comparisons can be made even when no items are strictly invariant across countries (Fox, 2010). Second, because the framework draws on random instead of fixed item effects, it presents a much more parsimonious representation of the differences among groups in terms of estimated model parameters. Investigations that include many groups are more feasible because the number of estimated parameters does not increase rapidly with the number of groups.

A nascent but growing body of research has demonstrated the potential of this approach (De Boeck, 2008; Muthén and Asparouhov, 2013). Simulation studies have shown that the multilevel random item effects framework recovers both overall and group-specific item parameters well in a variety of settings (Fox and Verhagen, 2010). Similarly, simulations assessing the comparative performance of invariance approaches have suggested that the approximate measurement invariance approach outperforms full and partial invariance approaches when there are many small differences in item parameters (Van de Schoot et al., 2013). Substantive applications have also emphasized the value of multilevel random item effects methods in accounting for response heterogeneity across groups (De Jong et al., 2008; Fox and Verhagen, 2010).

## Model formulation

When an IRM, such as those noted above, fit the data, we can separate estimates of the targeted latent trait from the distributional properties of items such that estimates generalize beyond the sampled observations and raters (Linacre, 1989). The critical assumption that allows for the separation of the latent trait from item characteristics is that measurement is invariant across subgroups of a population (Van de Schoot et al., 2013). Given a multilevel or cross-classified data structure, the conditions underlying the validity of this separation require invariance across each facet (e.g., participants, raters, observations).

More conceptually, construct-irrelevant variation can be split into two principal sources—latent trait side variation and item side variation. Latent trait side construct-irrelevant variation arises when the actual latent trait varies across design facets such as raters and/or observations. In contrast, item side variation arises when the underlying relationships between items and a latent trait vary across, for example, raters.

Under this division of construct-irrelevant variation, the aforementioned measurement models (Equations 1, 2) solely address latent trait variation across facets because they (only) decompose the variation in a latent trait into components uniquely attributable to each facet and do not address how item parameters vary across facets. Put differently, the latent trait side random effects models presented above account for the extent to which the latent trait of a participant is deflected by, for example, the relative severity of a rater and/or the atypical nature of an observation. In this way, latent trait side random effects models accommodate threshold differences among raters and observations only if these differences manifest consistently and uniformly for all items. If rather threshold differences among raters/observations vary across items or if discrimination parameters differ, latent trait side random effects models will not be sufficient to separate the latent trait from item characteristics because measurement is not invariant across facets.

Rather, in the presence of item side variance, separation of the latent trait from item characteristics would require direct treatment of measurement noninvariance. Applied to cross-classified rater-mediated assessments, conventional approaches, such as the partial invariance approach, are however particularly challenging because studies tend to draw on large number of raters and only a small number of items per latent trait. To relax assumptions of measurement invariance across raters, we developed a random item effects cross-classified (multilevel) graded response model. Our specification first drew on a graded response model parameterization such that observed item scores were treated as fallible ordinal ratings stemming from a targeted latent trait. Second, because many rater-mediated assessments operate within cross-classified (multilevel) designs, we leveraged a cross-classified (multilevel) graded response model to introduce random effects for distinct hierarchical units (e.g., raters). Third, we accommodated noninvariance across raters by permitting item discrimination and threshold parameters to vary across raters (and potentially another hierarchical unit) using random item effects (Fox, 2010). Under a repeated measures design, we express our model as

(4) $$\begin{array}{l} P(Y_{iotr}=k)=\Phi (a_{i}\theta_{t}+a_{ir}\alpha_{ot}+a_{ir}\gamma_{r}-d_{ir}^{k-1})\\ \;-\;\Phi (a_{i}\theta_{t}+a_{ir}\alpha_{ot}+a_{ir}\gamma_{r}-d_{ir}^{k}) \end{array}$$

Here *Y*_*iotr*_ is the ordinal score for item *i* in observation *o* for participant *t* rated by rater *r, a_*i*_* represents the average discrimination parameter for item *i* across all raters, θ_*t*_ represents a participant's persistent level of the targeted latent trait (i.e., across all observations), *a_*ir*_* is item *i*'s discrimination parameter under rater *r*, *α_*ot*_* is the latent trait deviation specific to observation *o* for participant *t*, and *γ_*r*_* is the deviation capturing consistent differences among raters in terms of their relative severity across all items. Let *K* represent the number of categories items are graded on with *k* as a specific category and let *d*^(1)^_*ir*_, …, *d*^(*K* − 1)^_*ir*_ be a set of *K* – 1 ordered item thresholds. That is, γ subsumes threshold differences among raters that are consistent across items, whereas *d* captures threshold differences among raters that are item-specific. To set the scale, let θ ~ *N*(0, σ^2^_*t*_), α ~ *N*(0, 1), γ ~ *N*(0, σ^2^_*r*_), *a*_*ir*_ ~ *N*(*a*_*i*_, σ^2^_*a*,*i*_), and *d*^*k*^_*ir*_ ~ *N*(*d*^*k*^_*i*_, σ^2^_*d*,*i*_).

In this particular specification, we used an independent random item effects structure and restricted item parameters to vary across only a single level two unit (raters). However, the model could be further extended to consider covariance among random item effects parameters and/or to allow item parameters to vary across both level two units (e.g., raters and participants). Similarly, we applied the mean item parameters across raters as the inter-rater item parameters and use these to construct an inter-rater scale. However, there are many reasonable and potentially more appropriate alternatives.

For instance, one alternative specification estimates the discrimination parameter applied to a participant's persistent level of the targeted latent trait (θ_*t*_) separate from the observation level discrimination parameter (*a_*ir*_*).

(5) $$\begin{array}{l} P(Y_{iotr}=k)=\Phi (a_{i}^{\left(t\right)}\theta_{t}+a_{ir}^{\left(o\right)}\alpha_{ot}+a_{ir}\gamma_{r}-d_{ir}^{k\;-\;1})-\Phi (a_{i}^{\left(t\right)}\theta_{t}\\ \;+\;a_{ir}^{\left(o\right)}\alpha_{ot}+a_{ir}\gamma_{r}-d_{ir}^{k}) \end{array}$$

Here we now use *a*^(*o*)^_*ir*_ as the observation level discrimination parameters (where *a*^(*o*)^_*ir*_ ~ *N*(*a*^(*o*)^_*i*_, σ^2^_*a*,*i*_)) and introduce *a*^(*t*)^_*i*_ as the participant level discrimination parameters which are nonrandom and unconnected to the observation level discrimination parameters. Under this specification, the scale of θ_*t*_ can be set by fixing its distribution to θ ~ *N*(0, 1).

The proposed model can also be adapted to accommodate other cross-classified or multilevel structures. For example, as noted earlier, many measurement designs operate within larger multilevel structures. Consider for example a design in which participants are cross-classified among raters and schools in which we track measurement noninvariance across raters. Under this design, the targeted latent trait of a participant now operates at lowest level of the hierarchy. With some slight changes in notation we can modify Equation (4) so that

(6) $$\begin{array}{l} P(Y_{itsr}=k)=\Phi (a_{i}\theta_{s}+a_{ir}\alpha_{ts}+a_{ir}\gamma_{r}-d_{ir}^{k\;-\;1})\\ \;-\;\Phi (a_{i}\theta_{s}+a_{ir}\alpha_{ts}+a_{ir}\gamma_{r}-d_{ir}^{k}) \end{array}$$

Here *Y*_*itsr*_ is the ordinal score for item *i* of participant *t* in school *s* rated by rater *r, a_*i*_* represents the average discrimination parameter for item *i* across all raters, θ_*s*_ represents the school effect or school-specific deviation in the latent trait, *a_*ir*_* is item *i*'s discrimination parameter under rater *r*, α_*ts*_ is participant *t*'s level of the targeted latent trait, and γ_*r*_ is the deviation specific to rater severity. Remaining notation and constraints are unchanged.

Our formulation of approximate measurement invariance models for rater-mediated assessments within a cross-classified (multilevel) structure is an extension of the multilevel IRM with random item effects (Fox, 2007). The proposed method first conceptualizes rater-mediated assessments and differential item functioning across raters within a multilevel random item effects framework. In turn, the method extends strictly hierarchical structures to accommodate cross-classified data structures where level one units (e.g., observations) are simultaneously nested within two independent level two units (e.g., raters and participants). Subsequently, we used this cross-classified framework to introduce hierarchically defined latent variables for both the targeted construct and the items to capture their respective variability across distinct level two units.

As noted earlier, construct-irrelevant variation can be conceptually split into two principal sources—latent trait and item side variation. Latent trait random effects (e.g., Equations 1, 2) serve to decompose the variation in a latent trait across facets. In contrast, item side random effects serve to capture the extent to which items function differently across hierarchical units. By simultaneously introducing latent trait and item side random effects, we permit a latent trait to vary across hierarchical units and items to function differently across those hierarchical units. When the proposed model fits the data, decomposing the latent trait and adjusting for differential item functioning across raters through random effects can establish an inter-rater scale such that the latent trait is separable from construct-irrelevant variation. In this way, estimates of a targeted latent trait from models that accommodate both latent trait and item side variation are more likely to generalize beyond the sampled observations and raters.

The key addition in the approach is the introduction of item side random effects across raters within a cross-classified framework. Random item effects are intended to not only identify noninvariance but also to track it through empirical estimates of the differences among raters. Under a Bayes approach, empirical estimates of rater-specific differences in item parameters are obtained using a mix of the inter-rater item parameters, which are based on all observations, and rater-specific item parameters, which are based on the particular observations a rater has rated. Rater-specific differences in item parameters are estimated using a shrinkage estimator where the amount of shrinkage toward the inter-rater estimates is a function of how precisely we can identify raters' differences from the mean. In this way, random item effects allow us to borrow strength from the larger pool of raters to improve estimates for individual raters, especially those for which we have little information. The shrinkage of rater-specific item parameters toward inter-rater parameters has been shown to reduce the mean-squared error of rater-specific estimates and is widely used elsewhere (Lindley and Smith, 1972; Raudenbush and Bryk, 2002; Fox, 2010).

In situating the proposed repeated measures model (Equation 4) among more conventional models, a single level IRM assumes that associations among items derive solely from a targeted latent trait. A multilevel IRM with observations nested within participants (ignoring raters) suggests that associations among items derive from a persistent component of a targeted latent trait and observation-specific deviations. Use of a cross-classified IRM with observations cross-classified among raters and participants suggests associations among items are a function of a persistent component of a targeted latent trait, observation-specific deviations, and deflections due to consistent differences in severity among raters. In these latent trait side (only) random effects models, item parameters are assumed to remain equal across raters. If we further introduce random item effects into the cross-classified model (Equation 4), we relax this assumption of equality of item parameters across raters and allow the discrimination and threshold parameters to vary.

### Estimation

The cross-classified structure of this model combined with the potential for a large number of latent variables renders maximum likelihood estimation computationally challenging with even a few items because it would require high dimensional numerical integration. A more practical option in this context is Bayesian methods (Gelman et al., 2004; Fox, 2007; Asparouhov and Muthén, 2012). Albert and Chib (1993) described a Gibbs sampler for a graded response model by using normally distributed latent item responses, *Z_*iotr*_*. Under this formulation, an observed ordinal response, *Y*_*iotr*_, is used as an item of a normally distributed latent item response, *Z_*iotr*_*, which is placed into a response category defined by threshold parameters *d*^*k*^_*ir*_ such that *Z_*iotr*_* is defined as

(7) $$\begin{array}{l} Z_{iotr}|Y_{iotr}=k,\theta_{t},\alpha_{ot},\gamma_{r},d_{ir}^{k},d_{ir}^{k\;-\;1},a_{ir},a_{i}\sim N(a_{i}\theta_{t}\\ \;+\;a_{ir}\alpha_{ot}+a_{ir}\gamma_{r},1)I(d_{ir}^{k\;-\;1}<Z_{iotr}\leq d_{ir}^{k}) \end{array}$$

This framework and its variations have been extended to incorporate multilevel structures and can be implemented in, for example, Mplus (De Jong et al., 2007; Asparouhov and Muthén, 2010a,b; Fox, 2010; Muthén and Muthén, 1998–2012).

### Testing for noninvariance

Having introduced random item effects to accommodate measurement noninvariance across raters, a relevant question is how we might test for evidence of (non)invariance. If measurement invariance holds, the variance of the random item effects across raters should be zero (e.g., σ^2^_*a*,*i*_ = 0). That is, if the variance of the random item effects is zero, item parameters are consistent across raters and measurement is invariant. However, departures from zero for specific items suggest that measurement is noninvariant across raters because the relationship between an item and the latent trait is not consistent across raters.

To examine evidence for measurement invariance and assess relative model fit, we can employ Bayesian tests of measurement invariance (Verhagen and Fox, 2013). These tests evaluate the variance components of the random item effects by using the Bayes factor to compare the ratio of the marginal likelihood of the null model (invariance) with the marginal likelihood alternative (noninvariance). Within the context of random item effects models, Bayesian tests of measurement invariance can be used to test invariance for each item parameter simultaneously by comparing models estimated with a diffuse prior against those using an informative prior concentrated at zero (e.g., inverse gamma distribution with a small scale parameter). Such comparisons potentially identify differential item functioning and directly assess the extent to which the fit of a model with fixed item parameters is improved upon by allowing item parameters to vary. Additional tests of, for example, factor variance invariance can also be investigated (e.g., Steenkamp and Baumgartner, 1998).

## Application

To probe the potential value and utility of the proposed methods, we applied our proposed model to a study of teaching quality using repeated classroom observations of mathematics teaching. As noted earlier, we investigated three questions focused on (a) evidence of noninvariance, (b) improvements in relative and absolute fit, and (c) improved predictive validity. Although we use this application as an initial case study of the proposed method, we are cautious to note that the correct underlying model is unknown because it is an empirical investigation. For this reason, the extent to which differences among approaches represent true gains or the extent to which these gains might be generalizable is unknown and needs to be studied further.

### Data description

In assessments of teaching quality, classroom observations of teaching are generally carried out by having trained raters evaluate teachers across multiple observations using a fixed set of items. Teaching evaluation instruments typically focus raters' attention on behaviors that exemplify an implicit theory of effective teaching. For each item, the guiding rubric that accompanies each instrument typically provides specific examples and descriptive anchors for each category of a scale and raters typically provide ordinal assessments for each item in each observation.

Like other types of rater-mediated assessments, a significant source of construct-irrelevant variation in classroom observations is differences among raters in their judgments (Kane and Staiger, 2012). The issue of rater differences can be especially pronounced in modern classroom observation systems because, unlike their historical counterparts, modern systems go beyond simple low inference checklists and rely more on inferential judgments. Recent investigations have demonstrated that even with extensive rater training, substantial differences among raters persist (Bell et al., 2012; Hill et al., 2012; Kane and Staiger, 2012).

Our data on teaching quality came from the National Center for Teacher Effectiveness study, which focused on identifying teacher characteristics and teaching practices that correlate with teacher effects as measured through student test score outcomes. Data for this analysis focus on classroom observations across two academic years of 150 fourth- and fifth-grade mathematics teachers and their students situated within across four large urban school districts in the Eastern United States. Each observation lasted about an hour and teachers were observed over three different occasions across an academic year. For each of these occasions, teachers were rated using the Mathematical Quality of Instruction (MQI) classroom observation system (Hill et al., 2008).

#### Teacher quality measure

The MQI observation system is a subject-specific observation instrument that was designed to provide a balanced view of mathematics instruction (Hill et al., 2008). In the current investigation, we focused our analyses on a general teaching quality domain which was captured using four ordinal items. The first item measured the extent to which the observed classroom work was consistently and directly connected to mathematics content (CWCM). The second item, richness of the mathematics instruction (RICH), captured the depth of the mathematics offered to students (Hill et al., 2008). The third item, Working With Students (WWS), captured the quality with which teachers understand and respond to students' mathematically substantive productions. The final item measured student participation in meaning-making and reasoning (SPMMR). This item captured students' involvement in cognitively demanding tasks and the extent to which students participated in and contributed to meaning-making and reasoning.

For each observation, raters independently evaluated teachers' instruction along each of the items by grading them on an ordinal scale ranging from a low of one to a high of three according to the descriptive anchors provided by the MQI rubric. The only exception was the CWCM item which was dichotomous. As a result, evaluations for each observation consisted of ordinal scores on a fixed set of items with each observation cross-classified by two hierarchical grouping structures—teachers and raters.

Each of the 39 raters in this study completed an online MQI training program (approximately 16 h) and then passed a subsequent certification exam. Raters also completed weekly calibration exercises where their scores were compared to master scores on clips of instruction. These scores were discussed in weekly webinars with master raters to help prevent rater drift. Raters who demonstrated problematic scores or rationales were remediated by master raters.

#### Student achievement measure

To measure student achievement, we used a researcher developed test administered to students in all four districts during the fall and spring semesters of the 2010–11 and 2011–12 school years. Items on this low-stakes mathematics assessment were designed to align with fourth and fifth grade Common Core mathematics standards, and covered topics such as numbers and operations, algebra, and geometry and measurement. Reliability of the test ranged from 0.82 to 0.89, depending on the form (Hickman et al., 2012).

To measure the average student achievement gains associated with each teacher in our sample, we estimated the following hierarchical linear model.

(8) $$a_{j,t,f}=A_{j,t,f-1}\pi +X_{j,f}\beta +\xi +\mu_{t}+\zeta_{t,f}+\varepsilon_{j,t,f}$$

The outcome variable, *a*_*j*, *t*, *f*_, represents the performance on the mathematics assessment of student *j* taught by teacher *t*, at time *f*. The model conditioned on a vector of prior achievement measures, *A*_*j*, *t*, *f* − 1_, which includes a cubic polynomial term for prior achievement on the same assessment^2^, a standardized English assessment, and their classroom aggregates; time varying demographic indicators, *X*_*j*, *f*_, for student *j* at time *f* (which include race, gender, subsidized-lunch eligibility, English language learner status, and special education status; and indicators for district, grade, and year of the assessment, ξ); and residual effects for the teacher (μ_*t*_), time(ζ_*t*, *f*_), and student (ε_*j*, *t*, *f*_). To estimate the underlying teacher effect or “value-added” score, we used the empirical Bayes residual for each teacher.

### Method

We applied the previously described random item effects cross-classified graded response model (Equation 4). We estimated the models in Mplus using the default diffuse prior distributions (see Appendix). Prior distributions for the discrimination parameters were normal with mean zero and variance five; for the thresholds the prior distributions were normal with mean zero and infinite variance, and for the variance parameters the prior distributions were log uniform bounded by negative and positive infinity. Subsequent inferences were conducted on the posterior medians and standard deviations. For each model, we ran two chains using a burn-in of 25,000 MCMC iterations and up to 100,000 post-burn-in iterations with convergence determined by the default potential scale reduction criteria implemented in Mplus and Gelman-Rubin diagnostics (Gelman and Rubin, 1992).

To assess evidence of measurement noninvariance, we first examined the variances of the item effects and their posterior distributions. To further appraise evidence for measurement invariance and assess relative fit, we employed the aforementioned Bayesian tests of measurement invariance for the null hypothesis that the variance of each item parameter was zero. To do so, we re-estimated the random item effects models using an inverse gamma (informative) prior with a shape parameter value of one and a scale parameter value of 0.005. We then explored the absolute fit using simple posterior predictive checks (Gelman et al., 2004). Finally, we evaluated the predictive capacity of the models by correlating teaching quality with value-added scores. Throughout the analyses we compared the results of the random item effects cross-classified graded response model with the results of alternative models which assume measurement invariance to assess the potential differences across models.

### Results

Table 1 presents the posterior item parameter estimates (on a probit scale) from a single level, a multilevel (occasions nested within teachers), a cross-classified (occasions nested within teachers and raters), and a random item effects cross-classified graded response models (Equation 4). For each model without random item effects, we present the item parameters and their uncertainty as captured by the posterior standard deviation. For the model which incorporates random item effects, we include the inter-rater item parameters and the uncertainty of those means using the posterior standard deviation. In addition, we summarize the variability of the item parameters across raters and 95% posterior intervals because the distributions of variance estimates are frequently skewed.

**Table 1:** **Discrimination and threshold parameters**.

	**Single**		**Multilevel**		**Cross-classified**		**Random item effects cross-classified**				
**Parameter**	**Est**	***SD***	**Est**	***SD***	**Est**	***SD***	**Est**	***SD***	**Item variance across raters**	**Low**	**High**
**DISCRIMINATION (*a_*i*_*)**											
RICH	1.14	0.04	1.08	0.04	0.99	0.05	1.05	0.07	0.10	0.05	0.20
WWS	1.39	0.07	1.18	0.06	1.15	0.05	1.46	0.11	0.19	0.08	0.44
CWCM	0.79	0.06	0.78	0.07	0.76	0.06	0.74	0.09	0.08	0.02	0.21
SPMMR	1.33	0.06	1.23	0.05	1.17	0.06	1.16	0.07	0.11	0.05	0.23
**THRESHOLD (*d_*i*_*)**											
RICH(1)	0.61	0.03	0.72	0.07	0.74	0.12	0.56	0.12	0.12	0.06	0.24
RICH(2)	2.57	0.06	2.88	0.08	2.93	0.14	2.71	0.13			
WWS(1)	0.53	0.04	0.57	0.04	0.64	0.13	0.48	0.15	0.07	0.01	0.24
WWS(2)	2.75	0.10	2.80	0.07	2.94	0.14	3.12	0.20			
CWCM(1)	−1.98	0.06	−2.24	0.12	−2.25	0.15	−2.39	0.15	0.09	0.02	0.25
SPMMR(1)	0.83	0.04	0.94	0.08	1.03	0.16	0.83	0.13	0.25	0.13	0.49
SPMMR(2)	2.78	0.09	3.06	0.12	3.24	0.19	2.97	0.15			
**LATENT TRAIT VARIANCE**											
Observations	1.00	—	1.00	—	1.00	—	1.00	—			
Teachers	—	—	0.34	0.05	0.40	0.06	0.32	0.06			
Raters	—	—	—	—	0.28	0.09	0.26	0.09			

Est, estimate; SD, standard deviation; Item Variance Across Raters, the item-specific random effect variance across raters (σ^2^_*a*,*i*_, σ^2^_*d*,*i*_); Low and High, the lower and upper bounds of the 95% posterior interval respectively.

The results of the random item effects model suggested that the item discrimination and threshold parameters varied across raters and thus were noninvariant (Table 1). Based on their posterior distributions, 95% posterior intervals suggested that the variance of their discrimination and threshold parameters was significantly different than zero. When the magnitude of item side variation across raters for each item is placed alongside the variance of the latent trait attributable to raters, the results suggested item side variation for each item was about half as large. That is, the variance in the latent trait across raters was about 0.26 (see last row of Table 1) whereas the average variance of item parameters among raters across all items was 0.13 (average of item variances in Table 1).

To put this into context, consider the Richness item. The estimated variance implies that although the item discrimination parameter was on average about 1.05 across all raters, the discrimination parameter for this item varied depending on who rated an observation (Table 1). For a rater who is two standard deviations above average, the estimated discrimination parameter could be as high 1.67 (using double the square root of the “Item Variance Across Raters” column in Table 1). In contrast, a rater who is two standard deviations below average, the estimated discrimination parameter for the same item could be as low as 0.43.

**Figure 1:**
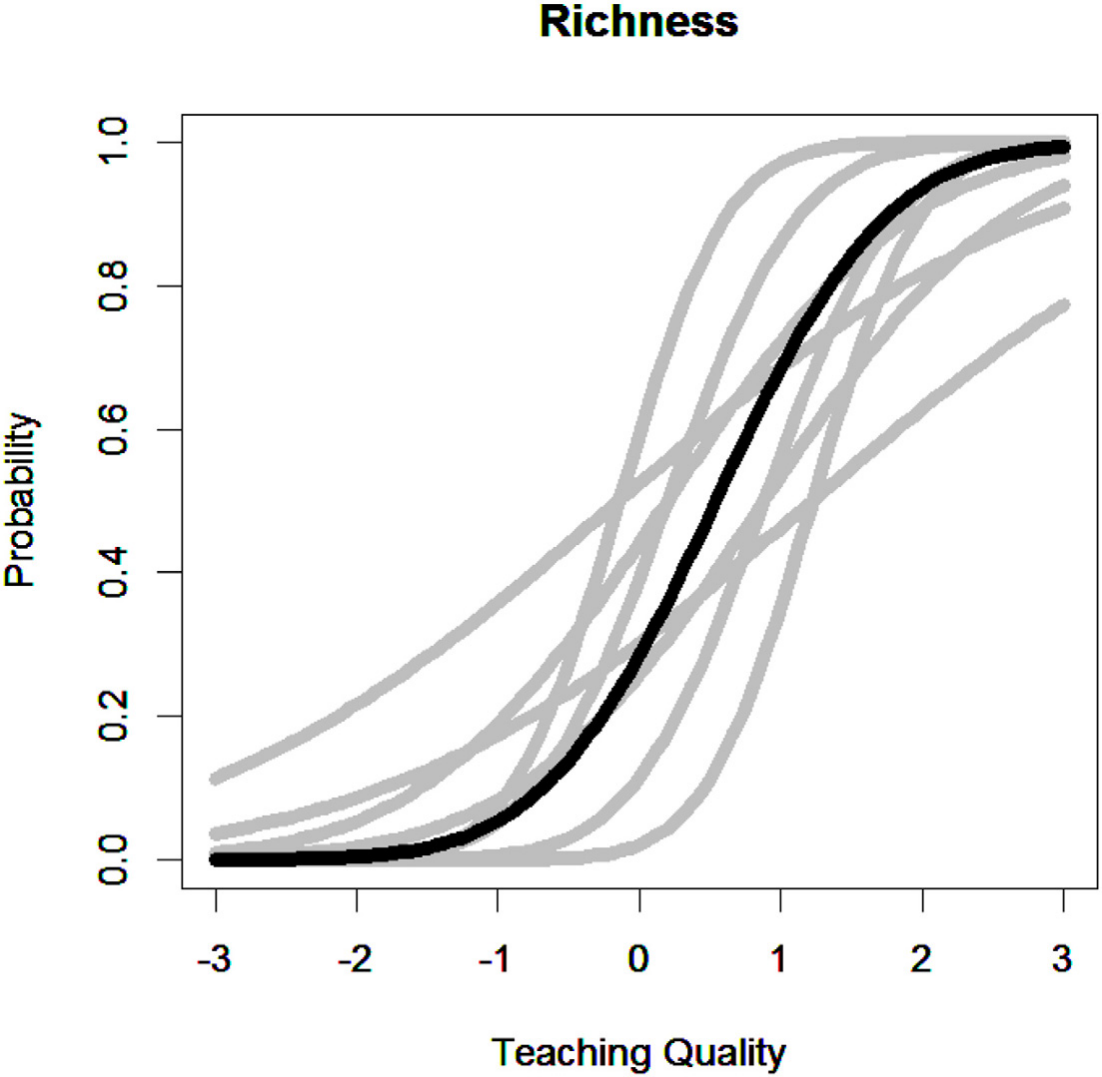
**Item characteristic curve for a single item across different raters**.

To illustrate the implications of this noninvariance, Figure 1 describes the item characteristic curves across raters for the richness item for the first threshold. In this figure, the dark curve represents the inter-rater item characteristic curve which is the average across all raters. In contrast, the gray curves describe the item characteristic curves for raters who are approximately one or two standard deviations above or below the average discrimination and threshold estimates for this item. Evident from this figure, which rater rates an observation has important implications for the scale of ratings and the extent to which teachers are placed on a similar scale.

To formally test measurement invariance across raters for each item and to assess relative fit, we re-estimated the random item effects model using an inverse gamma prior distribution of *IG*(1, 0.005) for the variance of each item parameter to test the null hypotheses that each of the variances was less than 0.001, 0.01, or 0.1. Using a common cutoff of about three for Bayes factor, the results for each threshold and discrimination parameter uniformly indicated that the variance of the random effects was different than zero (Jeffreys, 1961). In Table 2, we present the estimated variance along with the bounds of its 95% credible intervals and the Bayes factors for each item parameter under the hypotheses that the respective variance is less than 0.001, 0.01, or 0.1.

**Table 2:** **Test of measurement invariance for item parameters**.

**Parameter**	**Variance**	**Low**	**High**	***BF* < 0.001**	***BF* < 0.01**	***BF* < 0.1**
**THRESHOLD**						
RICH	0.12	0.06	0.24	0.000	0.000	0.576
WWS	0.07	0.01	0.24	0.623	0.668	1.009
CWCM	0.09	0.02	0.25	0.053	0.276	0.884
SPMMR	0.25	0.13	0.49	0.000	0.000	0.009
**DISCRIMINATION**						
RICH	0.10	0.05	0.20	0.000	0.000	0.858
WWS	0.19	0.08	0.44	0.000	0.000	0.353
CWCM	0.08	0.02	0.21	0.172	0.256	0.986
SPMMR	0.11	0.05	0.23	0.000	0.001	0.783

BF, Bayes factor for each item parameter under the hypotheses that the respective variance is less than 0.001, 0.01, or 0.1; Low and High, the lower and upper bounds of the 95% posterior interval respectively.

We further examined the fit of the models using posterior predictive checks for items. Overall, we found little difference across models. Table 3 contrasts the observed probability for each category by each item with the model based predicted probability for each model. In each case, the model largely recovers the observed probabilities. The multilevel model slightly misestimated probabilities for the RICH and CWCM items, the cross-classified model without random item effects slightly misestimated the RICH and SPMMR items, and the random item effects cross-classified model slightly misestimated the CWCM item.

**Table 3:** **Posterior predictive checks for item fit (95% posterior intervals)**.

**Item category**	**Observed**	**Single level**		**Multilevel**		**Without random item effects**		**With random item effects**	
		**Low**	**High**	**Low**	**High**	**Low**	**High**	**Low**	**High**
RICH0	0.656	0.649	0.663	0.648	0.692	0.637	0.709	0.618	0.69
RICH1+	0.344	0.337	0.351	0.308	0.352	0.291	0.363	0.31	0.382
RICH2	0.044	0.042	0.047	0.031	0.043	0.027	0.042	0.034	0.052
WWS0	0.622	0.613	0.629	0.604	0.65	0.597	0.676	0.573	0.656
WWS1+	0.378	0.371	0.387	0.35	0.396	0.324	0.403	0.344	0.427
WWS2	0.053	0.05	0.057	0.043	0.058	0.038	0.059	0.045	0.071
CWCM0	0.060	0.058	0.062	0.042	0.052	0.041	0.061	0.035	0.05
CWCM1	0.940	0.938	0.942	0.948	0.958	0.939	0.959	0.95	0.965
SPMMR0	0.691	0.683	0.698	0.678	0.723	0.676	0.749	0.668	0.738
SPMMR1+	0.309	0.302	0.317	0.277	0.322	0.251	0.324	0.262	0.332
SPMMR2	0.047	0.044	0.05	0.034	0.048	0.027	0.044	0.03	0.047

To further contrast the methods, we examined the correspondence of their teaching quality estimates. We first examined the correlation among scores from alternative methods. Results indicated that estimates from alternative methods were correlated with the proposed method between 0.89 and 0.93 (Table 4). Next, we considered the discrepancy among implied teacher classifications. Current and forthcoming policy often requires that teachers be stratified into about four categories (e.g., Hansen et al., 2013). For each set of scores we classified teachers into quartiles and identified the percentage of discrepant classifications. Results indicated that discrepancy rates between the proposed method and the alternative methods were relatively high and ranged from 23 to 37% (Table 5). Put differently, based on a sample size of 150 teachers, approximately 35 to 56 would be classified differently across methods.

**Table 4:** **Correlation among observation scores from different methods**.

**Method**	**RIE-CC**	**CC**	**ML**	**Single**	**Averages**
RIE-CC	1.00	0.93	0.91	0.90	0.89
CC	0.93	1.00	0.92	0.91	0.92
ML	0.91	0.92	1.00	0.96	0.95
Single	0.90	0.91	0.96	1.00	0.99
Averages	0.89	0.92	0.95	0.99	1.00

RIE-CC, random item effects cross-classified graded response model; CC, cross-classified graded response model; ML, multilevel graded response model; Single, single level graded response model

**Table 5:** **Discrepant classification rates among methods**.

**Method**	**RIE-CC**	**CC**	**ML**	**Single**	**Averages**
RIE-CC	0.00	0.23	0.32	0.37	0.33
CC	0.23	0.00	0.26	0.30	0.32
ML	0.32	0.26	0.00	0.24	0.23
Single	0.37	0.30	0.24	0.00	0.09
Averages	0.33	0.32	0.23	0.09	0.00

RIE-CC, random item effects cross-classified graded response model; CC, cross-classified graded response model; ML, multilevel graded response model; Single, single level graded response model.

As noted earlier, a primary benchmark for the validity of classroom observations is their efficacy in predicting student achievement gains. To examine our final research question, we investigated the extent to which allowing item parameters to vary across raters improved the predictive validity of the teaching observation scores as compared to more conventional approaches. To get a sense of the extent to which improvements in predictive validity were attributable specifically to random item effects across raters, we examined correlations for models that sequentially introduced key features. Table 6 displays the correlations between teachers' value-added scores and their teaching quality estimates from the single level, multilevel, cross-classified, and random item effects cross-classified models.

**Table 6:** **Correlation between observations scores and value-added scores**.

	**Estimate**	**Low**	**High**
Averages	0.11	−0.05	0.27
Single	0.12	−0.04	0.28
Multilevel	0.15	−0.01	0.31
CC	0.14	−0.02	0.30
RIE-CC	0.17^*^	0.01	0.33

* Interval excludes zero.

RIE-CC, random item effects cross-classified graded response model; CC, cross-classified graded response model; ML, multilevel graded response model; Single, single level graded response model.

The results suggested gains as models increasingly took into account integral features of classroom observation data. Using simple averages, the correlation between observation and value-added scores was 0.11. By applying item response theory and acknowledging the ordinal nature of the scale, this correlation increased by about 10%. By introducing random observation effects through a multilevel model to account for the dependence of items within an observation, the correlation increased an additional 30%. In contrast, further introducing a random effect for raters through a cross-classified model (but restricting item parameters to be invariant across raters), decreased the correlation by about 10%. However, once we allowed for random item effects, the cross-classified model again increased the correspondence between observation and value-added scores. Moreover, although 95% intervals for the correlation between observation and value-added scores included zero across models without random item effects, the 95% interval for the correlation excluded zero for the model with random item effects.

## Discussion

Although strict measurement invariance across raters is optimal, the reality is that it will rarely hold in rater-mediated assessments. Developing measurement models that are more tightly attuned to the types of measurement errors present in rater-mediated assessments is likely to improve the validity and comparability of scores across raters and other sources of construct-irrelevant variation. The proposed method relaxes assumptions of measurement invariance in cross-classified (multilevel) rater-mediated assessments by introducing random item effects to test for noninvariance and empirically construct an inter-rater scale. More conceptually, the approach helps to identify the “ruler” each rater uses to conduct his/her assessments, construct an inter-rater scale, and make adjustments to observed scores in order to place them on this inter-rater scale.

Evidence from the case study on teaching quality suggested the promise of random item effect models in addressing noninvariance in rater-mediated assessments. The results indicated that measurement was noninvariant across raters for each item and suggested that direct adjustments for this noninvariance through random item effects improved model fit and the predictive validity of the teaching quality. These results are consistent with prior literature in that they suggest that ignoring measurement noninvariance can obscure both the psychometric properties of a scale and the underlying relationships among variables.

As noted previously, the results presented in this study are only based on a single case study and do not necessarily imply these findings will generalize. However, although the authority of the proposed model over alternative models is unclear in our empirical application, the more flexible assumptions of the proposed model with regard to measurement noninvariance would seem to lend greater credence to its results. Nevertheless, the circumstances under which the proposed method outperforms alternative methods need to be systematically studied in greater detail to understand the extent to which findings are robust to key assumptions.

In this regard, we highlight four areas that warrant further study. First, the flexibility of the proposed framework suggests many different alternative forms and we have presented just a few limited forms. For instance, we chose to define inter-rater parameters as the average of item parameters and apply those values to the teacher level construct. However, there are many reasonable alternatives including not linking parameters at hierarchical levels to those at the lower level at all and independently estimating them. Future research will need to investigate alternatives, develop tests for comparing the fits of non-nested models, and examine the extent to which results are robust to these choices.

Second, in our application we assumed random item effects were independently normally distributed. For our case study, *post-hoc* analyses examining the tenability of the normality assumption for each item parameter using the Shapiro–Wilks test of normality were conducted. Each test suggested that we could not reject the null hypothesis that the random item effects came from a normal distribution. However, this assumption may be untenable if, for example, items are invariant across most raters but demonstrate substantial invariance for a handful of raters. In this case fixed multiple group approaches are potentially more appropriate. Similarly, its reasonable to suspect that random item effects may not be independent. In *post-hoc* analyses we re-estimated the proposed model using a multivariate normal distribution for the random item effects. Our results indicated virtually no correlation among the random effects. However, for many assessments, its reasonable to suspect that a rater who is above average at discriminating on one item may also be above average at discriminating on other items.

Third, having established noninvariance, an important follow-up question examines the extent to which rater characteristics systematically predict noninvariance. For example, do raters with more years of experience demonstrate a greater capacity to discriminate among quality levels? To address this line of inquiries, the proposed model can be further extended to include explanatory components such that random item effects are modeled as a function of fixed rater characteristics through a latent regression framework (De Boeck and Wilson, 2004).

Fourth, the results of our case study suggested that adjustment for persistent differences in severity among raters actually decreased the correspondence between observation and value-added scores. More specifically, when we compared the results of the multilevel model that did not adjust for rater effects at all with that of the cross-classified model with rater severity adjustments (but no random item effects), the correlation between teaching and value-added scores decreased (see Multilevel vs. CC in Table 6). These differences could be spurious but they raise questions concerning the value of uniform adjustments for rater severity. In another *post-hoc* analysis, we re-estimated the random item effects cross-classified model (Equation 4) but omitted the overall adjustment for rater severity (γ_*r*_). Our results indicated that absolute fit remained the same but that the correlation between observation and value-added scores increased to 0.20. Again, although the authority of these differences is unknown, these results question the conventional wisdom of including broad sweeping and uniform adjustments for rater severity. Future investigations should examine the fidelity of such adjustments and further consider the efficacy of interactions among the facets. For instance, literature has found that raters function differently across subgroups so that they are more severe within certain subgroups than others.

In conclusion, meaningful comparisons among participants on latent traits in rater-mediated assessments require measurement to be invariant across raters. In many instances, this assumption will be unrealistic. The proposed method offers a flexible alternative that can accommodate measurement noninvariance within multilevel and cross-classified frameworks even when there are no invariant items. Our results suggest the approach is promising and flexible but that it needs more investigation.

### Conflict of interest statement

The authors declare that the research was conducted in the absence of any commercial or financial relationships that could be construed as a potential conflict of interest.

## Footnotes

^1^We use the term “item” to describe indicators of a latent trait in a broad sense.

^2^One district did not take the study distributed assessment in the fall semester of school year 2010–11 (pretest), so we used student performance on the state standardized math exam in its place for this prior achievement control.

## Appendix

### Example Mplus code

TITLE: Random item effects;

DATA: FILE IS data.dat;

VARIABLE: NAMES ARE tid rid rich wws cwcm spmmr;

CATEGORICAL = r ich wws cwcm spmmr;

CLUSTER = rid tid;

ANALYSIS: ESTIMATOR = BAYES;

TYPE = CROSSCLASSIFIED RANDOM;

Process = 2;

MODEL:

%WITHIN%

s1-s4 | fw by orich* owws cwcm ospmmr;

fw@1;

%BETWEEN TID%

ft BY orich* owws cwcm ospmmr (p1-p4);

s1-s4@0;

%BETWEEN RID%

[s1-s4] (p1-p4);

fw;
